# Supplementary material for: Antidiabetic Effect of Millet Bran Polysaccharides Partially Mediated via Changes in Gut Microbiome
Source: Foods. 2022 Oct 28;11(21):3406. doi: 10.3390/foods11213406 (PMC9654906; doi:10.3390/foods11213406)
Supplement: Supplementary file 1 [file foods-11-03406-s001.zip › foods-1976301-supplementary.pdf]

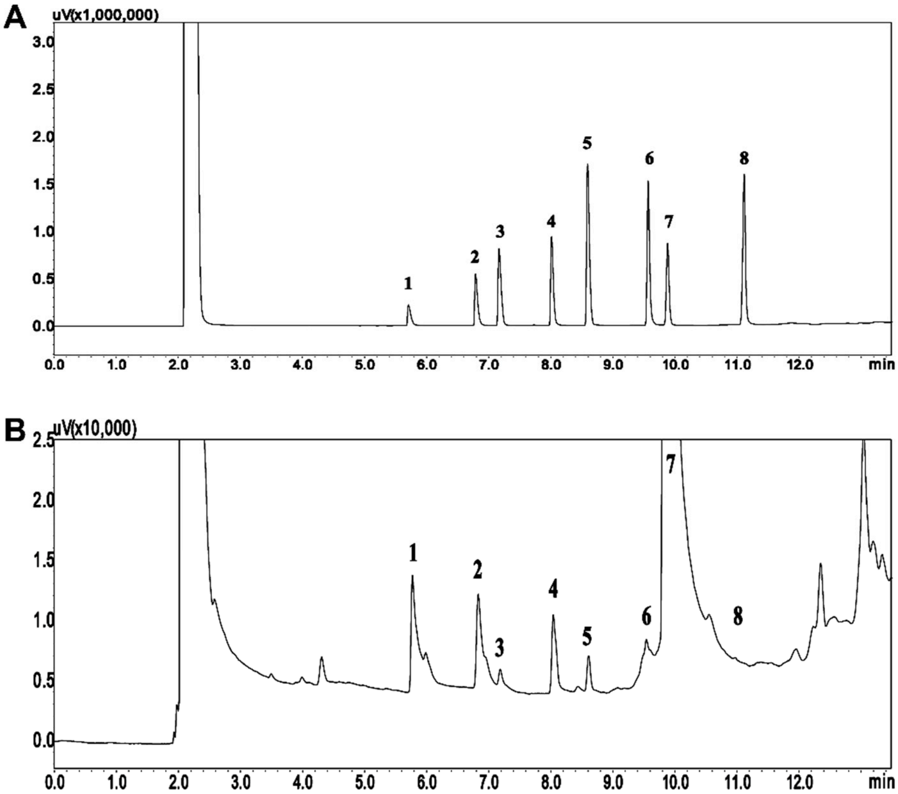

Figure S1. Standard curve (A) and samples curve (B) of SCFAs.

Table S1. Standard curves of SCFAs

Group	Rt/min	Standard Curve	R^2
AA	5.71	y = 0.0099x + 0.0023	0.9905
PA	6.789	y = 0.0219x + 0.0061	0.9903
iBA	7.167	y = 0.0339x + 0.0128	0.9923
BA	8.013	y = 0.0341x + 0.0128	0.9918
iVA	8.594	y = 0.0644x + 0.0238	0.9920
VA	9.569	y = 0.0528x + 0.0159	0.9902
CA	11.115	y = 0.0589x + 0.0086	0.9905
